# Evidence for panmixia despite barriers to gene flow in the southern African endemic, *Caffrogobius caffer *(Teleostei: Gobiidae)

**DOI:** 10.1186/1471-2148-8-325

**Published:** 2008-12-01

**Authors:** Marlene Neethling, Conrad A Matthee, Rauri CK Bowie, Sophie von der Heyden

**Affiliations:** 1 Evolutionary Genomics Group, Department of Botany and Zoology, Stellenbosch University, Private Bag X01, Matieland 7602, South Africa; 2 Museum of Vertebrate Zoology and Department of Integrative Biology, 3101 Valley Life Science Building, University of California, Berkeley, USA

## Abstract

**Background:**

Oceanography and life-history characteristics are known to influence the genetic structure of marine species, however the relative role that these factors play in shaping phylogeographic patterns remains unresolved. The population genetic structure of the endemic, rocky shore dwelling *Caffrogobius caffer *was investigated across a known major oceanographic barrier, Cape Agulhas, which has previously been shown to strongly influence genetic structuring of South African rocky shore and intertidal marine organisms. Given the variable and dynamic oceanographical features of the region, we further sought to test how the pattern of gene flow between *C. caffer *populations is affected by the dominant Agulhas and Benguela current systems of the southern oceans.

**Results:**

The variable 5' region of the mtDNA control region was amplified for 242 individuals from ten localities spanning the distributional range of *C. caffer*. Fifty-five haplotypes were recovered and in stark contrast to previous phylogeographic studies of South African marine species, *C. caffer *showed no significant population genetic structuring along 1300 km of coastline. The parsimony haplotype network, AMOVA and SAMOVA analyses revealed panmixia. Coalescent analyses reveal that gene flow in *C. caffer *is strongly asymmetrical and predominantly affected by the Agulhas Current. Notably, there was no gene flow between the east coast and all other populations, although all other analyses detect no significant population structure, suggesting a recent divergence. The mismatch distribution suggests that *C. caffer *underwent a population expansion at least 14 500 years ago.

**Conclusion:**

We propose several possible life-history adaptations that could have enabled *C. caffer *to maintain gene flow across its distributional range, including a long pelagic larval stage. We have shown that life-history characteristics can be an important contributing factor to the phylogeography of marine species and that the effects of oceanography do not necessarily suppress its influence on effective dispersal.

## Background

With no obvious physical barriers to gene flow, marine species with large ranges are expected to show genetic homogeneity over long stretches of ocean – a panmixia paradigm [1]. Many studies have, however provided evidence to the contrary, finding strong genetic structure and detecting oceanic barriers to gene flow that often correspond to biogeographic regions [2,3]. It is now known that several factors, including oceanography [4,5], life history traits [6] and contemporary factors such as local adaptation [[6] and references therein] may strongly influence the genetic structuring of marine organisms despite the passive dispersal opportunities that the marine environment offers.

**Figure 1:**
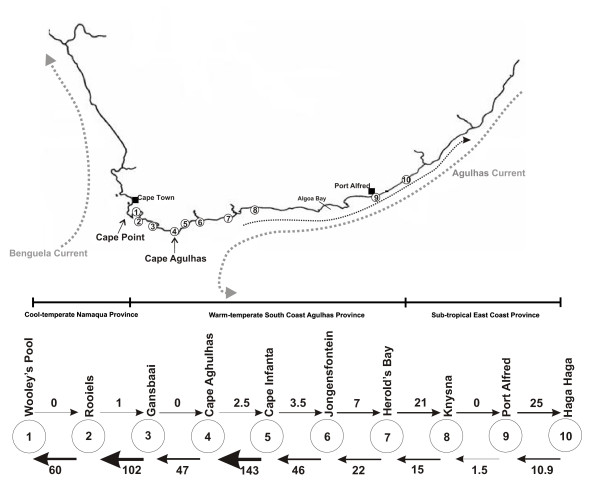
**Map of South Africa showing sampling localities for *Caffrogobius caffer***. The two major currents, the Benguela and Agulhas currents are shown, as well as the smaller inshore counter-current to the Agulhas. 1 = Wooley's Pool in False Bay; 2 = Rooiels; 3 = Gansbaai; 4 = Cape Agulhas; 5 = Cape Infanta; 6 = Jongensfontein; 7 = Herold's Bay; 8 = Knysna; 9 = Port Alfred; 10 = Haga Haga. The stepping stone model shows the directionality and relative intensity of gene flow between populations using arrows. The three major marine biogeographic regions in southern Africa are also delineated.

The South African coastline offers a unique setting to study the relative importance of oceanography on the genetic structuring of marine organisms. There are two contrasting current systems that meet as the continental shelf widens between Cape Agulhas and the Cape Peninsula (Figure 1). These are the warm Agulhas Current, which flows southwards from Mozambique along the eastern coast of the country, and the cold Benguela Current that runs northwards along the western coast [7]. The Agulhas Current deflects away from the coastline near Algoa Bay, resulting in mixing of warm and cool water around the South Coast [[7,8]; Figure 1]. Based on the distribution patterns of currents, topographical features and species compositions [9], three biogeographical regions are recognized along the South African coastline. These are the subtropical East Coast (from KwaZulu-Natal to east of Algoa Bay), the warm-temperate South Coast (from Algoa Bay to Cape Point), and the cold-temperate West Coast (from Cape Point to the Namibian border) [[9]; Figure 1].

It has traditionally been accepted that species with good dispersal abilities should show little or no genetic structure over large stretches of ocean, whereas live-bearers and species with short larval durations (or larval retention strategies) should be more strongly structured genetically [10]. However, many closely related species with the same potential dispersal abilities have been found to show contrasting phylogeographic patterns over the same geographic range [11,12]. In addition, data derived from 70 studies that investigated the population genetic structure of species across the Atlantic-Mediterranean break have recently been re-analyzed and it has been suggested that in this region there may not be a relationship between biological traits and phylogeographic structure of marine species [3]. In South Africa, all intertidal and shallow subtidal species studied to date have exhibited a genetic break over Cape Agulhas (the southernmost point of Africa), irrespective of their dispersal abilities [13-17]. Furthermore, larval duration, swimming abilities and retention strategies (e.g. brooding of eggs) only seem to influence the phylogeography of South African invertebrates on a regional scale (within biogeographic regions), as even the mudprawn (*Upogebia africana*) and caridean shrimp (*Palaemon peringueyi*) with long-lived, actively swimming larvae exhibit at least one genetic break [15].

*Caffrogobius caffer *is a goby endemic to South Africa. These fish are abundant in rock pools and primarily restricted to the high intertidal zone. As such they are well adapted to survive extreme temperature and salinity ranges [[18]; authors pers. obs]. They have been cited as occurring from False Bay (Cape Peninsula) on the south-west Coast to Delagoa Bay in southern Mozambique [19], although Hoese [20] states that these gobies do not occur in Mozambique and we, despite intensive sampling, did not catch any east of Haga Haga on the east coast. Importantly, mark-and-recapture studies strongly suggest that adult movement is limited to nearby pools only [18], making adult dispersal over long distances highly unlikely. The reproductive biology of *C. caffer *is not well documented, but insights from the closely related *Caffrogobius gilchristi*, an estuarine inhabitant, could be used as an indicator. However, the duration of their pelagic larval stage can only be speculated on, compared with findings for closely related species (Strydom, pers. comm.). It is also likely that male gobies guard their eggs until they hatch.

Our objective was to determine whether life history influences the population structure of a widely distributed fish species that is primarily restricted to life in the high intertidal zones. Given the length of coastline investigated (~1300 km) and that *C*. *caffer *probably has limited dispersal abilities due to an abbreviated life history where male fish guard the eggs, we expect to find evidence of population genetic structuring along the distribution range, particularly over the regions strongest barrier to gene flow, Cape Agulhas. Further, we hypothesised that gene flow patterns are strongly influenced by the dominant current systems in the range of *C. caffer*, in particular by the Agulhas Current, and accordingly, we should find asymmetric gene flow patterns between populations. Further, we aim to elucidate whether life-history characteristics are indeed unimportant predictors of genetic structure of South Africa's coastal biota [13-16], i.e. that oceanography is the dominating force in shaping populations in the region.

## Methods

### Sampling protocol and laboratory procedures

Fishes were collected from ten localities covering a wide distributional range of the species using ethylenglycolmonophenylether (phenoxyethanol; Merck) added to rockpools and handnets (Figure 1). Sampling was carried out within the laws of South Africa. Despite intensive sampling, we could not obtain samples from the central and northern Kwazulu-Natal coastline where *C. caffer *seems to be replaced by *C. natalensis*. All fishes have been deposited in the JLB Smith collection at the South African Institute for Aquatic Biodiversity, Grahamstown. A small piece of muscle tissue was digested overnight in an extraction buffer containing 20 μl ProteinaseK at 55°C. DNA was extracted using the DNeasy™ Tissue Kit (Qiagen), following the protocol for animal tissues. Primers and PCR cycling conditions of Lee *et al. *[21] were used for amplification of the 5'-end of the mitochondrial DNA control region; a marker that has proved useful in numerous population genetic studies, especially of fishes. PCR products were gel purified using the Illustra™ DNA and Gel Purification Kit (GE Healthcare). Cycle sequencing was carried out using BigDye Terminator chemistry (Applied Biosystems) and the products were analysed on an automated sequencer (AB 3100, Applied Biosystems).

### Statistical analyses

Sequences were edited and aligned by eye with BioEdit ver7.0.4.1 [22]. Sequences were checked against goby control region sequences on GenBank, with a tblastx search, to ensure that the correct region was amplified. Haplotypes were identified in Collapse1.2 http://darwin.uvigo.es. Haplotype (*h*) and nucleotide diversity (*π*) indices were calculated for each population individually and for the whole dataset, in Arlequin3.1 [23]. All haplotypes have been deposited in [GenBank: EU335086–EU335140].

#### Population structure

Analysis of Molecular Variance (AMOVA) was carried out in Arlequin3.1 to investigate the geographical distribution of haplotypes and provide the fixation indices for between- and within population variation. Φ_*ST *_values were calculated to take the number of mutational changes between the haplotypes into consideration [24]. SAMOVA was used to calculate the geographic distribution of genetic structure. This maximises the proportion of the total genetic variation between groups of populations, as well as identifying possible genetic barriers between them, without pre-defining populations as is necessary for AMOVA [25]. The geographical coordinates of each sampling location were obtained from Google Earth^®^. To detect possible isolation by distance, a Mantel test was performed in Arlequin3.1 using 10 000 permutations and the measured geographical distances between localities determined from a scaled map as the shortest land-sea interface distance, but excluding bays.

#### Demographic history

Fu's F_*S *_test [26] was used to test for possible population expansion, for the whole dataset, as well as for each sampling location separately. A mismatch distribution [27] was also calculated to further investigate the demographic history of *C. caffer *populations. The Sum of Squared deviations and Harpending's Raggedness index were calculated to test for the goodness of fit of the data in the mismatch analyses. A parsimony minimum spanning network was constructed using TCS1.21 [28], which is a more appropriate way than constructing bifurcating phylogenetic trees to view the evolutionary relationship between closely related haplotypes where ancestry may not be strictly bifurcating [29].

The time of expansion was calculated with the formula T = τ/2 μ, where μ = generation time × number of base pairs per sequence × mutation rate for the marker used [30], and τ was calculated in Arlequin3.1. Although the generation time of *C. caffer *is not known, estuarine fishes (including *C. gilchristi*) are said to disperse to the marine environment to join spawning adults after one to three years residence time in the estuary [31]. Estuarine spawning does; however, occur from time to time [31], and therefore it is reasonable to assume a generation time of between one and three years for calculations of divergence times. A mutation rate of 3.6% per million years is generally used for teleosts [32], although the fast evolving control region of the mtDNA molecule has been found to have a mutation rate of 11–13% per million years in the white sturgeon [33]. Based on this, two separate calculations were done – one using a 3.6% per million years mutational rate and one based on a 11% per million years mutational rate.

#### Migration between sampling localities

To estimate past migration rates, as well as the directionality of gene flow between populations, Migrate-n version 2.4 was used [34,35]. In order to maximize the statistical power of our gene flow analyses we sought to minimize the number of parameters [see e.g. [36]] and constructed a stepping-stone model with asymmetrical gene flow. Our study system is particularly suited to this model, given the linear nature of the southern African coastline. For the stepping-stone migration model, two analytical runs were conducted, an initial short run, followed by a second longer run. For both runs, the starting values of the population mutation parameter and the ratio between the immigration rate and the respective population and mutation rate per generation were estimated from F_*ST *_values [34]. For the long-run 10 short-chains, each with a total of 25,000 generations and a sampling increment of 20 generations, and two long-chains each with a total of 250,000 generations and a sampling increment of 50 generations were run twice. A total of 50,000 and 12,500,000 genealogies (recorded steps multiplied by the sampling increment) were visited by the short and long chains, respectively. For both, the short and long chains, the first 10,000 genealogies were discarded (the burnin). An adaptive heating scheme with four chains (starting values of 5.00, 2.50, 1.50, 1.00) and a swapping interval of one was used to ensure that efficient mixing occurred. For the other settings, default values were implemented.

## Results

Between 21 and 26 fishes were obtained from each location; a total of 242 individuals (Table 1). After editing and alignment by eye in BioEdit, 390 base pairs remained for analyses. There were 26 polymorphic nucleotide positions (6.67% variable sites), with 20 observed transitions and eight transversions. Fifty-five haplotypes were recovered, of which 28 were restricted to single individuals. Haplotype diversity (*h*) was found to be very high (*h *= 0.956, ± 0.004) and nucleotide diversity (π), was low (π = 0.010, ± 0.005). Interestingly, theta, as estimated by MIGRATE [34,35] was lowest for the two peripheral populations at Wooley's Pool and Haga Haga.

**Table 1:** Population specific diversity indices of *Caffrogobius caffer *localities.

Sampling location	*n*	No. private haps.	No. haplotypes	*π*	*h*	Θ	**Fu's F**_*S*_
Wooley's Pool	22	3	12	0.008	0.948	0.0073	-7.0, p = 0.005
Rooiels	22	1	18	0.011	0.965	0.0186	-8.5, p < 0.001
Gansbaai	25	4	17	0.010	0.967	0.0122	-10.8, p < 0.001
Cape Agulhas	26	1	15	0.010	0.963	0.0141	-7.6, p = 0.001
Cape Infanta	26	5	17	0.010	0.969	0.0103	-14.4, p < 0.001
Jongensfontein	26	3	16	0.009	0.960	0.0116	-7.9, p < 0.001
Herold's Bay	26	1	15	0.009	0.951	0.0103	-8.0, p < 0.001
Knysna Heads	24	3	17	0.011	0.982	0.0104	-12.9, p < 0.001
Port Alfred	24	3	12	0.009	0.935	0.0117	-6.0, p = 0.002
Haga Haga	21	4	18	0.010	0.971	0.0074	-9.5, p < 0.001

*n *= sample size, *π *= nucleotide diversity and *h *= haplotype diversity.

### Population structure

Pairwise Φ_*ST *_values among sampled localities were low and all but one of these comparisons were not significant (Jongensfontein and Port Alfred, Φ_*ST *_= 0.046, *P *< 0.05). SAMOVA analyses maximized at two groups with Jongensfontein grouped separately from the rest of the sampling sites, although this grouping was not biologically meaningful as Jongensfontein is in the middle of the distribution range (F_*CT *_= 0.022, *P *< 0.05). No significant relationship between genetic and geographical distance was detected by the Mantel test (0.072, *P *> 0.05).

### Demographic history

**Figure 2:**
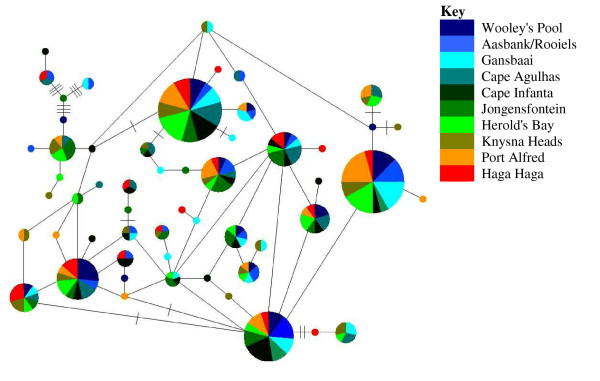
**Haplotype network for *C. caffer***. Size of the circles are representative of the number of individuals with that haplotype. The smallest circles represent a haplotype frequency of one. Each connecting line represents one mutation step between haplotypes and perpendicular lines are representative of an additional mutational change.

Fu's F_*S *_test for all sites was highly negative and significant (Table 1), as would be expected for populations that have undergone recent demographic change [37]. The mismatch distribution did not differ significantly from the null model of sudden expansion, with the observed and expected curves both indicative of population expansion (data not shown). The parsimony haplotype network constructed recovered no clear phylogeographic structure, indicating panmixia (Figure 2). There are two dominant haplotypes, each being present in 24 individuals (~10% of individuals), with representatives from all three biogeographic regions. Although all populations were characterized by between one and five private haplotypes (Table 1), extensive haplotype sharing occurred between the West Coast, South Coast and East Coast of the southern African coastline.

When using τ = 3.859 and the 11% per million years mutation rate for the mtDNA control region [33], the time of population expansion is estimated to be between 15 000 (3 year generation time) and 45 000 (1 year generation time) years ago, or between 45 800 and 138 000 years ago when estimated with a more conserved mutation rate of 3.6% per million years [32].

### Migration between sampling localities

Interestingly, coalescent analyses of gene flow revealed strong asymmetry along the distributional range of *C. caffer *(Figure 1, Table 2). There was limited gene flow from western to eastern populations, with strong gene flow from east to west, suggesting an important influence of the Agulhas Current. Notably, there is limited gene flow between Port Alfred and Haga Haga, the two most eastern populations and all other sampling sites, suggesting that they are becoming effectively isolated.

**Table 2:** Relative migration rate values (*N*_*m*_) between each population pair for the stepping-stone migration model along the South African coast.

From Population	To population	***N***_*m*_
1 (Wooley's Pool)	2 (Rooiels)	0 (0–10)
2 (Rooiels)	1 (Wooley's Pool)	60 (37–91)
2 (Rooiels)	3 (Gansbaai)	1 (0.06–4.5)
3 (Gansbaai)	2 (Rooiels)	201 (145–270)
3 (Gansbaai)	4 (Cape Agulhas)	0 (0–4)
4 (Cape Agulhas)	3 (Gansbaai)	47 (34–61)
4 (Cape Agulhas)	5 (Cape Infanta)	2.5 (0.7–5.5)
5 (Cape Infanta)	4 (Cape Agulhas)	143 (112–178)
5 (Cape Infanta)	6 (Jongensfontein)	3.5 (2–5)
6 (Jongensfontein)	5 (Cape Infanta)	46 (37–56)
6 (Jongensfontein)	7 (Herold's Bay)	7 (5–8.5)
7 (Herold's Bay)	6 (Jongensfontein)	22 (18–26)
7 (Herold's Bay)	8 (Knysna)	21 (18–24)
8 (Knysna)	7 (Herold's Bay)	15 (12–17)
8 (Knysna)	9 (Port Alfred)	0 (0-0)
9 (Port Alfred)	8 (Knysna)	1.5 (0.7–2.5)
9 (Port Alfred)	10 (Haga Haga)	25 (14–39)
10 (Haga Haga)	9 (Port Alfred)	39 (31–59)

Values in brackets represent the 0.05% confidence values.

## Discussion

### Demographic history

The mitochondrial control region proved to have sufficient variability for population genetic analysis. The combination of high haplotypic diversity and comparatively low nucleotide diversity is typical of the signature of population expansion after a bottleneck or a founder event [37-40], which is generally associated with little or no population structure [3,40]. Both the mismatch distribution and the significant Fu's F_*S *_(-25.6, *P *< 0.001) support that for *C. caffer *both as a species and at the individual population level has undergone recent population expansion (Table 1). The time of expansion is indicative of a growing population since the late Pleistocene (275 000 to 15 000 years ago, depending on the mutation rate and generation time used). Interestingly, similar estimates for expansion time have been found in other South African marine organisms, for example the lobsters *Palinurus delagoae *[41]*Palinurus gilchristi *[42] and *Jasus tristani *[43], as well as a hake species, *Merluccius capensis *[40], and it is likely that changing sea levels and temperatures during this time contributed to demographic changes.

Interestingly, although the two populations at the edge of *C. caffer *distribution also show signals of population expansions, their theta (4*N*_*e*_*mμ*) values are lower than when compared to all other sampling sites. This may be a signal of an expanding population into these areas or alternatively may reflect source-sink dynamics at the putative range edge of this species.

### Population structure and gene flow patterns

SAMOVA failed to identify any biologically meaningful groups and AMOVA revealed panmixia between populations of all three biogeographic regions. Further, isolation by distance [44] should not be expected in species with planktonic larvae that can successfully disperse over great distances and that are able to exchange individuals between the populations at the edges of their distribution ranges [45]. In concordance with this theory, significant isolation by distance was only found for South African organisms with possible larval retention or direct development and not for those with pelagic larvae [14-16] including *Caffrogobius caffer*. As it is highly unlikely that adult *C. caffer *disperse over large distances, the only possible dispersal option is that by larvae.

Interestingly, despite the signatures of a mtDNA panmictic population of *C. caffer *in South Africa, coalescent analyses of gene flow show that there is very limited gene flow (west to east *N*_*m *_= 0 (0,0); east to west *N*_*m *_= 1.5 (0.7, 2.5) between east coast (Port Alfred and Haga Haga) and all other populations (Figure 1, Table 2), suggesting that the east coast is effectively isolated. This is congruent with gene flow patterns in the clinid fish *Clinus cottoides *[17], where analyses also recovered no gene flow to or from the east coast populations. This is probably a function of distance between suitable rocky shore habitats. As one moves towards the east coast of South Africa, rocky shore habitats become progressively more sparse, with large interspersing tracts of sandy beaches and it probably becomes much less likely that larvae are able to migrate between populations. One possible reason for the absence of significant population structure between the east coast and all other populations is that the reduction in gene flow may be a geologically recent event, and that not enough time has passed to fix any subsequent population specific mutations at the mtDNA level.

Notably, the Agulhas Current plays a large role in the migration patterns of these fishes. There is little gene flow from west to east, even between closely situated populations. In contrast, extensive gene flow occurs from the east to the west coast, which is in the direction of the flow of the Agulhas Current. Some fishes have been shown to utilise the inshore counter-current that runs up the east coast (sardines; [8] and *Clinus cottoides*, [17]), yet this is not evident for *C. caffer*. This may be as a result of a longer pelagic larval stage, which allows *C. caffer *to disperse with the Agulhas Current, unlike fishes which either actively seek to use the counter-current or that have a very short pelagic larval stage.

Remarkably, these results contrast sharply to what has been found for rocky shore and shallow intertidal organisms in South Africa to date, which have shown population genetic structuring irrespective of their life-history patterns and pelagic larval duration [13-17]. Our most surprising finding is that gene flow is taking place across Cape Agulhas, which has been identified as major oceanographic barrier to gene flow in all intertidal and shallow water inhabitants studied to date [13-15,17]. Based on these findings it has been stated that oceanography strongly influences the dispersal potential of marine organisms in South Africa, possibly more so than having a pelagic larval stage [15,16]. Our results suggest that *C. caffer *has better dispersal abilities than expected (based on mark-recapture studies of adults) and that this may overcome the effects of such major oceanographic barriers.

### Dispersal capabilities of *Caffrogobius caffer*

Having the capability to release a large amount of eggs into the open ocean at the best possible time should greatly increase the chance of effective dispersal between populations, as in theory it only takes one (practically 5–10) successful migrant per generation to maintain some gene flow between populations [45]. In South Africa, larvae of members of the Gobiidae are numerically dominant in the nocturnal ebb tide – a synchronized hatching strategy used by many estuarine fishes to promote dispersal into the ocean [31,46]. *Caffrogobius gilchristi *in particular can potentially release millions of larvae into the open ocean during a single tidal cycle, thereby increasing their dispersal success [46]. As larvae of the genus *Caffrogobius *have not to date been identified to species level for all six species in the genus, it is not possible to know how numerically dominant *C. caffer *is in the surfwater (Strydom, personal communication). Judging from its panmictic mtDNA pattern though, we hypothesise that the species most probably is as successful as *C. gilchristi *in terms of reproductive output and dispersal. Our findings are suggestive of *C*. *caffer *having a longer larval duration than expected, for it is highly unlikely that individuals could be exchanged between the East Coast and South-west Coast within a matter of days if utilizing the large-scale Agulhas current system.

Together with larval duration, larval behaviour may also play an important role in the dispersal abilities of *C. caffer*, like it does in many other species [e.g. [4,47]]. Larvae of cryptobenthic fishes, including individuals from the family Gobiidae, usually possess good swimming and orientation abilities at time of settlement [48,49], which may greatly alter the expected population connectivity of the species [50]. Since post-flexion larvae in South African waters are known to be excellent swimmers [46], the wide dispersal of *C. caffer *larvae in local current systems seems probable.

## Conclusion

Our results show that successful dispersal across major oceanographic barriers such as Cape Agulhas is possible for some marine species and that availability of habitat is another key determinant in population structuring of marine organisms along the South African coast. We have shown that life-history characteristics could in fact be an important contributing factor to the population genetic structuring of marine species in southern Africa and illustrate that the influence on the effective dispersal of marine organisms is not necessarily suppressed by the effects of oceanography as has recently been advocated [3,15,16].

## Authors' contributions

MN carried out the majority of the field and laboratory work and wrote the draft version of the manuscript. CAM participated in field work, helped with the interpretation of data and finalisation of the manuscript. RCKB collected samples, helped with statistical analyses and contributed to writing the manuscript. SvdH conceived the study, helped with sampling and data analysis and contributed to writing the final manuscript. All authors have read and approved the final manuscript.
